# Benchmark maps of 33 years of secondary forest age for Brazil

**DOI:** 10.1038/s41597-020-00600-4

**Published:** 2020-08-14

**Authors:** Celso H. L. Silva Junior, Viola H. A. Heinrich, Ana T. G. Freire, Igor S. Broggio, Thais M. Rosan, Juan Doblas, Liana O. Anderson, Guillaume X. Rousseau, Yosio E. Shimabukuro, Carlos A. Silva, Joanna I. House, Luiz E. O. C. Aragão

**Affiliations:** 1 Tropical Ecosystems and Environmental Sciences lab – TREES, São José dos Campos, Brazil; 2 Instituto Nacional de Pesquisas Espaciais (INPE), São José dos Campos, Brazil; 3 University of Bristol, Bristol, United Kingdom; 4 Programa de Pós-graduação em Biodiversidade e Conservação, Universidade Federal do Maranhão (UFMA), São Luís, Brazil; 5 Laboratório de Ciências Ambientais, Centro de Biociências e Biotecnologia, Universidade Estadual do Norte Fluminense Darcy Ribeiro (UENF), Campos dos Goytacazes, Brazil; 6 University of Exeter, Exeter, United Kingdom; 7 Centro Nacional de Monitoramento e Alertas de Desastres Naturais (Cemaden), São José dos Campos, Brazil; 8 Programa de Pós-graduação em Agroecologia, Universidade Estadual do Maranhão (UFMA), São Luís, Brazil; 9 University of Maryland, College Park, United States of America; 10 University of Florida, Gainesville, United States of America

**Keywords:** Forest ecology, Climate-change mitigation, Ecosystem services

## Abstract

The restoration and reforestation of 12 million hectares of forests by 2030 are amongst the leading mitigation strategies for reducing carbon emissions within the Brazilian Nationally Determined Contribution targets assumed under the Paris Agreement. Understanding the dynamics of forest cover, which steeply decreased between 1985 and 2018 throughout Brazil, is essential for estimating the global carbon balance and quantifying the provision of ecosystem services. To know the long-term increment, extent, and age of secondary forests is crucial; however, these variables are yet poorly quantified. Here we developed a 30-m spatial resolution dataset of the annual increment, extent, and age of secondary forests for Brazil over the 1986–2018 period. Land-use and land-cover maps from MapBiomas Project (Collection 4.1) were used as input data for our algorithm, implemented in the Google Earth Engine platform. This dataset provides critical spatially explicit information for supporting carbon emissions reduction, biodiversity, and restoration policies, enabling environmental science applications, territorial planning, and subsidizing environmental law enforcement.

## Abstract (summary)

**Table:** 

Measurement(s)	secondary forest • age • Increment • Extent • secondary forest loss
Technology Type(s)	remote sensing • computational modeling technique • digital curation
Factor Type(s)	year of data collection
Sample Characteristic - Environment	tropical • forest ecosystem
Sample Characteristic - Location	Brazil

Machine-accessible metadata file describing the reported data: 10.6084/m9.figshare.12622025

## Background & Summary

**Fig. 1:**
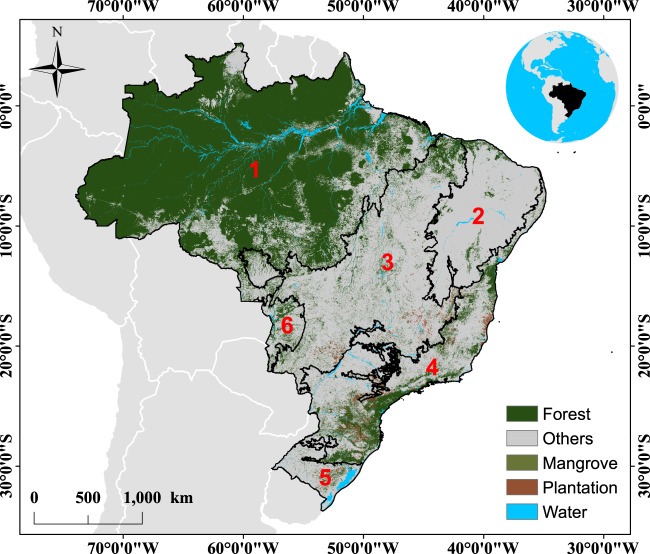
Forest cover of Brazil. In the main map, black lines represent the Brazilian biomes: 1. Amazon; 2. Caatinga; 3. Cerrado; 4. Atlantic Forest; 5. Pampa; 6. Pantanal. Source: land-use and land-cover map of 2018 from the MapBiomas Project (http://mapbiomas.org).

In Brazil (Fig. 1), forest cover (excluding mangroves and plantations) decreased from 4,646,516 km^2^ in 1985 to 4,079,827 km^2^ in 2018, a total reduction of 12% (566,689 km^2^) (https://mapbiomas.org; Collection 4.1)^1^; an area slightly larger than Spain. This forest loss depletes forest’s capacity to provide ecosystems services by reducing carbon and biodiversity stocks, as well as its water recycling potential, directly affecting climate and consequently, human populations^2–4^. While forest loss continues in Brazil at varying rates, secondary forests are regrowing on areas where old-growth forests have been completely removed by human disturbances^5^. The extent and age of Amazonian secondary forests have already been quantified and their spatial-temporal patterns are highly dynamic^6,7^. The long-term dynamics of Brazilian’s secondary forests is still poorly quantified. This knowledge, however, provides key information for assisting Brazil to achieve its intended Nationally Determined Contribution (NDC) targets agreed at the United Nations Framework Convention on Climate Change (UNFCCC) and for supporting the territorial planning required for compliance with the environmental legislation. Here we address this lack of information by producing and making available a set of annual secondary forest growth maps at 30-m spatial resolution from 1986 to 2018 at a national level. We stablished that secondary forest growth occurs when a pixel classified as anthropic cover^8^ (e.g., pasture or agriculture) in a given year is replaced in the following year by a pixel of forest cover (excluding mangroves and plantations).

Secondary forests are essential to mitigate climate change, as they are highly productive, with an average net carbon uptake rate for neotropical regions of 3.05 Mg C ha^−1^ yr^−1^, 11 times the rate of old-growth forests^9^. Secondary forest regrowth can also mitigate biodiversity loss, allowing the species pool to recover in Amazonia^10^. Species richness and compositional similarity of secondary forests reach on average 88% and 85%, respectively, of values found in old-growth forests after 40 years^10^. In Atlantic Forest fragments, secondary forest-growth recovered around 76% of taxonomic, 84% of phylogenetic and 96% of functional diversity over a period of 30 years after abandonment. Besides, the recovery of these fragments, when compared with primary forests, allowed the retrieval of 65% and 30% of threatened and endemic species, respectively^11^. Considering these benefits, the management of natural regeneration may be the most effective strategy to promote large-scale forest restoration^12–14^.

From 1996 to 2015 natural regeneration in the Atlantic Forest recovered 2.7 Million ha of forest cover, representing about 8% of the current forest cover (34.1 Million ha)^15^. In addition, this biome has an estimated potential for natural regeneration of 2.8 Million ha by 2035^15^. Indeed, the restoration and reforestation of 12 million hectares of secondary forests is one of the main mitigation strategies for reducing carbon emissions within the Brazilian NDC^16^. This instrument needs to be accompanied by political and economic incentives, necessary for conducting the transition from the current productive model based on extensive environmental degradation to an alternative model promoting the emergence of new secondary forests, as well as the maintenance of the remaining forests^17^. The latter, if well planned, can provide direct benefits to local economies and communities, incentivizing rural producers to preserve secondary forests^18^.

Thus, understanding the dynamics of secondary vegetation in the Brazilian territory is essential to mitigate the negative impacts of climate change, to avoid carbon and biodiversity loss, and to guide decision-makers in creating conservation policies for these forests fostering the sustainable development. In this data descriptor, we produced spatially explicit annual maps of secondary forest increment, extent, age, and loss, from 1986 to 2018 for the whole Brazil, at 30-m of spatial resolution. Hence, our dataset allows applications on local, regional or national scales favouring the emergence of studies providing data that are able to support policies focusing on functional landscape management aiming to promote restoration of secondary forest areas for the benefit of peoples’ lives.

## Methods

**Fig. 2:**
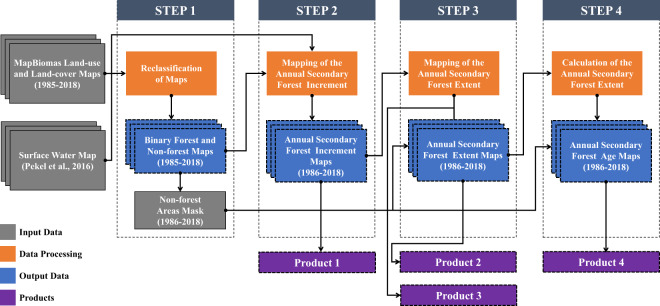
Workflow of the proposed method.

Our method was implemented in the Google Earth Engine (GEE) platform^19^. We divided it into four steps. Figure 2 summarizes our approach, including the input of the raw data (land-use and land-cover from 1985 to 2018 and the water surface), and the output data (from 1986 to 2018), which included maps of the annual secondary forest increment (Product 1), annual secondary forest extent (Product 2), annual secondary forest loss (Product 3; from 1987 to 2018), and annual secondary forest age maps (Product 4).

### Input data

We used the land-use and land-cover data from the Brazilian Annual Land-Use and Land-Cover Mapping Project (MapBiomas Collection 4.1; https://mapbiomas.org/en/colecoes-mapbiomas-1)^1^ as input data. This dataset was obtained through the classification of images from the Landsat satellite series (30-m spatial resolution) using a theoretical algorithm implemented in the GEE platform^19^. Details about the processing of the dataset can be found in the Algorithm Theoretical Basis Document^20^. More detail about the land-use and land-cover classes can be found in the MapBiomas website (https://mapbiomas.org/en/codigos-de-legenda?cama_set_language=en).

Moreover, we used the maximum water surface extent data (from 1984 to 2018) developed by Pekel *et al*.^21^ (https://global-surface-water.appspot.com) to avoid the inclusion of false detection within wetland areas in our products. This dataset contains a map of the spatial distribution of the water surface cover from 1984 to 2018, globally^21^. These data were obtained from 3,865,618 Landsat 5, 7, and 8 scenes acquired between 16 March 1984 and 31 December 2018. Each pixel was individually classified into water or non-water cover using an expert system^21^ implemented in the GEE platform^19^.

### Step 1 – Reclassifying MapBiomas data

All MapBiomas land-use and land-cover maps from 1985 to 2018 (34 maps) were reclassified into binary maps. We assigned the value “1” for all pixels in the Forest formation class of the MapBiomas product (Legend ID: 3) and “0” for the other land-use and land-cover classes. In our reclassified maps, pixels with value of “1” were, then, associated to the class “Forest”, which includes only forests classified as old-growth and secondary (before 1985). Mangrove and forest plantation classes were excluded from our secondary forest map.

### Step 2 – Mapping the Annual Increment of Secondary Forests

We mapped the annual increment of secondary forests using the forest maps produced in Step 1. This process was carried out pixel-by-pixel, where every pixel classified as Forest (value 1) in the analysed year (*y*_*i*_; between 1986 to 2018) and classified as non-forest (value 0) in the previous year (*y*_*i-1*_*; i = *1985, 1986… 2017) was mapped as secondary forest. As forest cover maps before 1985 were not available in the MapBiomas product, maps of secondary forest increment start in 1986, when it was possible to detect the first transition (1985 to 1986). Thus, 33 binary maps were obtained, where the secondary forest increments (non-forest to forest) have a value of 1 and the other transitions a value of 0 (forest to forest, non-forest to non-forest, and forest to non-forest). Here, we only considered secondary forest growth in pixels that had previously an anthropic cover (forest plantation, pasture, agriculture, mosaic of agriculture and pasture, urban infrastructure, and mining) and did not overlap wetland areas.

### Step 3 – Mapping the Annual Extent of Secondary Forests

**Fig. 3:**
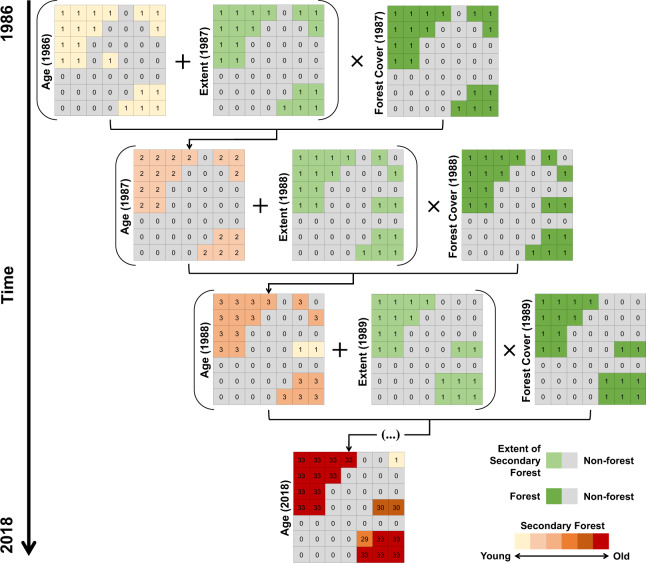
Conceptual model of the approach used to calculate the age of secondary forests throughout the Brazilian territory.

We generated 33 maps of the annual extent of secondary forests. To produce the map of secondary forest extent in 1987, we summed the map of the total secondary forest extent in 1986, which is the same map as the secondary forest increment in 1986 from step 2, with the 1987 increment map, resulting in a map containing all secondary forest pixels from 1986 and 1987. Knowing that the sequential sum of these maps results in pixels with values higher than 1, to create annual binary maps of secondary forest extent, we reclassified the map produced for each year by assigning the value 1 to pixels with values between 2 and 33 (secondary forest extent) and pixels with a value 0 were kept unchanged. Finally, to remove all secondary forest pixels that were deforested in 1987, keeping in the map only pixels with the extent of stand secondary forests, we multiplied the resulting map by the annual forest cover map of 1987, produced in step 1 (Fig. 3). This procedure was applied year-by-year from 1986 to 2018 to produce the maps of annual secondary forest extent. The removal of deforested pixels provides a product depicting the extent of secondary forest deforested in each specific year and they were also included as complimentary maps (from 1987 to 2018) in our dataset.

### Step 4 – Calculating the Age of Secondary Forest

**Fig. 4:**
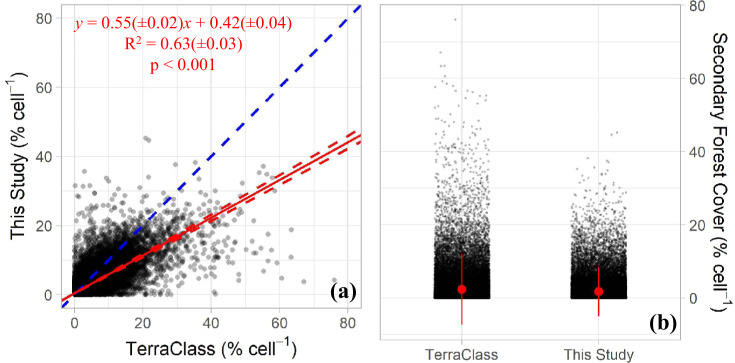
**(a)** Scatter-plot for the relationship between the proportion of the secondary forest within the 10 by 10 km cells in the two datasets. The dashed blue line is the 1:1 line; the red line is the average regression from the bootstrap approach with 10,000 interactions; the dashed red lines are regressions using the standard deviation values of the equation parameters. All p-values from the 10,000 bootstrap interactions were lower than 0.001. **(b)** Jitter-plot for the proportion of the secondary forest within the 10 by 10 km cells. The red dot is the mean, and the red vertical line the standard deviation.

Finally, we calculated the age of the secondary forests (Fig. 3). First, we summed the 1986 map of annual secondary forest extent (from Step 3) with the 1987 map to obtain the age of secondary forests in 1987 (Fig. 4). We continued this summation year-by-year until the secondary forest age map of 2018 was obtained (Fig. 4). The values of each pixel in 2018 correspond to the age of the secondary forest. To ensure the elimination of deforested secondary forests from each age map, we executed a similar procedure as described in step 3 by removing all forest pixels overlaying non-forest areas (Fig. 4). As our analyses started in 1986, it was not possible to identify secondary forests before this year. The 1986 age map, therefore, only shows one-year old secondary forests, and the 2018 map shows ages of secondary forest varying between 1 and 33 years (Fig. 4). If a secondary forest pixel with any age is cleared in a given year, it is then removed and a value of zero is attributed to the pixel. The age of this pixel, subsequently, will only be computed again if the algorithm detects a new non-forest to forest transition in the forest cover map (Step 1), which depends on the MapBiomas project classification method.

## Data Records

The dataset provides 33 maps of annual secondary forest age, 33 maps of annual secondary forest extent and 33 maps of annual secondary forest increment from 1986 to 2018 for the entire Brazil. Also, the dataset provides 32 maps for annual secondary forest loss from 1987 to 2018 for the entire Brazil. All maps are in Geographic Coordinate System with Datum WGS84, the same as the input dataset. The archive is available at Zenodo (10.5281/zenodo.3928660)^22^. The dataset contains the classified maps in compressed TIFF format (eight tiles per year; see https://github.com/celsohlsj/gee_brazil_sv) at 30-m spatial resolution, grouped in annual zipped files. The dataset can also be accessed through the Toolkit Download (available from https://github.com/celsohlsj/gee_brazil_sv). In the Toolkit Download, the data can be subset and exported (as compressed TIFF format) by administrative boundaries (states and municipalities), watersheds, biomes, and protected areas. The dataset will be updated as new MapBiomas collections become available.

## Technical Validation

This dataset was based on the Collection 4.1 of MapBiomas Project (Annual Land-Use and Land-Cover Maps of Brazil)^1^; thus, the accuracy of the secondary forest increment, extension and age maps presented here is anchored to the accuracy of the MapBiomas land-use and land-cover dataset. The MapBiomas analyses of accuracy were performed using the Pontius Jr and Millones (2011) method^23^. For the entire Brazil^24^, the MapBiomas dataset has an average of 86.40 ± 0.46% of overall accuracy, 11.06 ± 0.67% of allocation disagreement, and 2.5 ± 0.29% of area disagreement between 1985 and 2018, considering the land-use and land-cover classes from the legend level with the greatest detail (level 3).The accuracy assessment for the Brazilian biomes can be found in the MapBiomas accuracy statistics web page (https://mapbiomas.org/en/accuracy-analysis).

In addition to the MapBiomas validation, we also compared the secondary forest map from the method proposed here with the secondary forest map from the TerraClass project (Official Brazilian Amazon Map of Land-use and Land-cover). The TerraClass project provides maps of the land-use and land-cover of previously deforested areas in the Brazilian Amazon using independent methods, including supervised classification and visual interpretation of Landsat images and time-series analysis of images from the MODIS sensor^8^.

To perform the comparison, we only considered secondary forests that grew in the Brazilian Amazon between the 2005–2014 period and remained unaltered until 2014. To minimize any discrepancies related to methodological differences between the two methods, we restricted our analysis to only account for the geographical area monitored by the TerraClass project. Following the method proposed by Gasparini *et al*.^25^, we first calculated the proportion^26^ of secondary forest cover for the two maps using 43,293 regular 10 by 10 km grid-cells. Subsequently, the comparison between the two datasets was carried out using a bootstrap approach, implemented in R statistical software v.4.0.2 (R Core Team, 2020)^27^, with 10,000 interactions. For each interaction, the algorithm randomly raffled 10% of the 43,293 cells with replacement. Finally, based on the bootstrap results, we calculated the mean and standard deviation of the 10,000 coefficients of determination (R^2^), intercepts, slopes, and root mean squared errors (RMSE).

We found that the proportion of secondary forests mapped by our method explained on average 63 ± 3% (R^2^ = 0.63 ± 0.03 and RMSE = 2.04 ± 0.09%) of the TerraClass secondary forests proportion (Fig. 4a). The non-parametric Mann-Whitney test^28^ (Fig. 4b) showed that the mean of secondary forest proportion from our method (1.69 ± 3.35% cell^−1^) was significatively (W = 864,240,514 and p < 0.001) lower than the mean secondary forest proportion from TerraClass (2.32 ± 4.87% cell^−1^). Finally, in Table 1, we show the results of the Mann-Whitney test by intervals of the secondary forest proportion of the TerraClass. For all analysed intervals, the mean of secondary forest proportion from our method was significatively lower than the mean secondary forest proportion from TerraClass. In the dominant class of secondary forest proportion (0% to 10%), which represents 94.4% of the regular 10 by 10 km grid-cells analysed, the difference in the extent of secondary forests between the two products was very small (0.21%). Analysing the second most dominant class (11% to 20%) this difference increased to 5.8%. Despite statistically different, the resulting secondary forest proportion differences between the maps for the two dominant classes, which together account for 98.8% of all grid-cells analysed, indicates that our secondary forest cover map is consistent with the TerraClass product.

**Table 1:** The non-parametric Mann-Whitney test by TerraClass Cell Proportion Intervals.

Statistics		TerraClass Cell Proportion Intervals				
		0–10%	11–20%	21–30%	31–40%	41–80%
W		755,276,307	3,078,744	115,974	10,698	3,844
p		<0.001	<0.001	<0.001	<0.001	<0.001
n		40,881	1,898	348	104	62
%		94.40	4.40	0.80	0.20	0.10
Mean ± SD	TerraClass (% cell^−1^)	1.40 ± 2.48	14.65 ± 2.66	25.21 ± 2.82	35.43 ± 3.16	48.74 ± 7.66
	This Study (% cell^−1^)	1.19 ± 2.32	8.88 ± 4.94	13.84 ± 6.25	16.57 ± 6.87	16.97 ± 8.77

In the table, W is the Mann-Whitney test statistic, p is the p-value, n is the number of observations, % is the percentage of the total sample size in each class, and SD is the standard deviation.

## Usage Notes

Understanding the dynamics of secondary forests in the tropical region has always been a challenge. In Brazil, with its continental dimension, it is no different. Thus, freely available maps of the annual increment, extent, and age of secondary forest associated to complimentary maps of the annual deforestation of secondary forests, at high spatial resolution, are essential to support the implementation of forest restoration policies for biodiversity conservation and carbon emissions reduction. This dataset also enables the development of other environmental sciences applications, territorial planning, and environmental law enforcement activities.

**Fig. 5:**
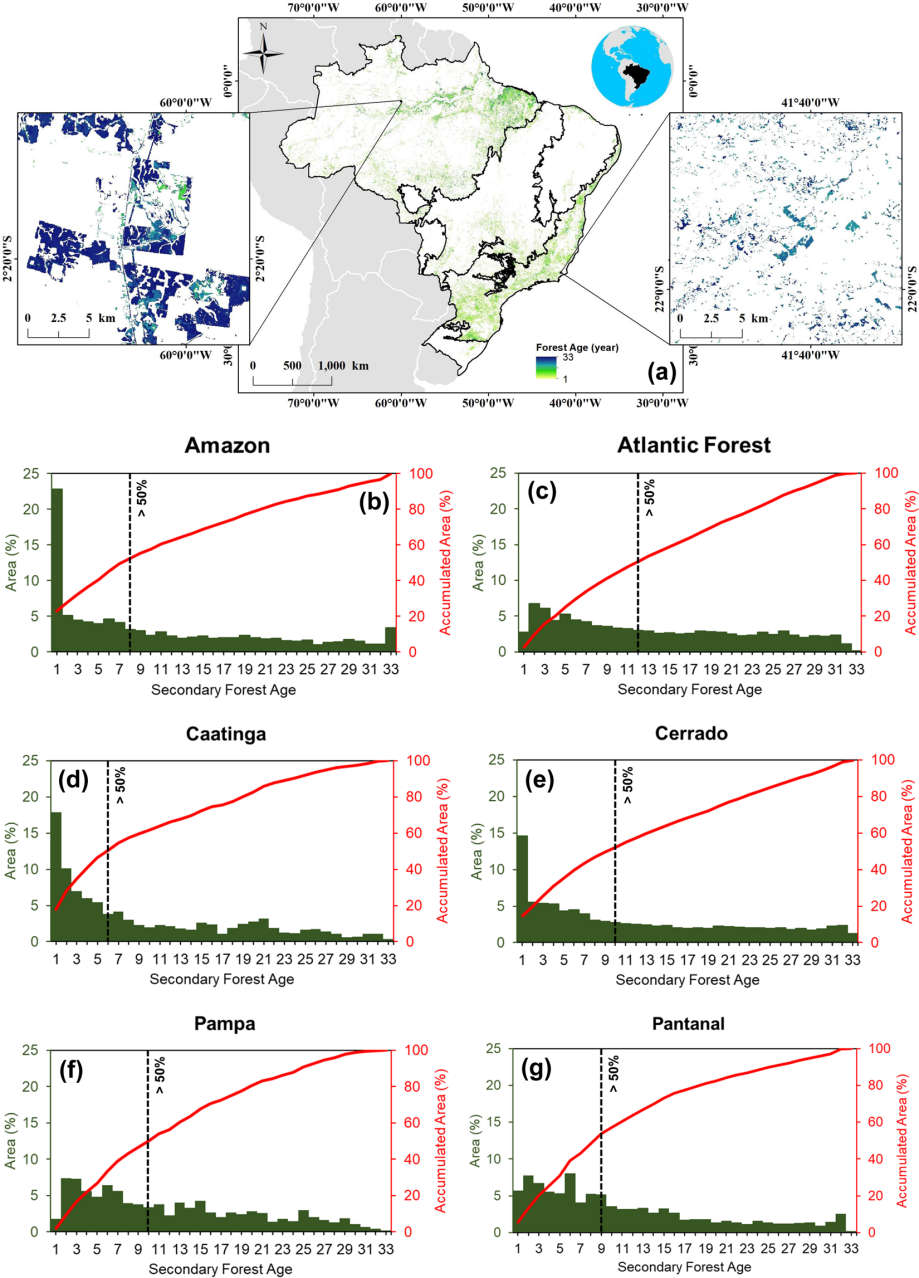
**(a)** Map of Brazil with secondary forests identified using the process outlined in the text. The detailed map on the left shows the age of secondary forests in the Amazon, while the detailed map on the right shows the age of secondary forests in the Atlantic Forest. **(b**–**g)** Histogram of secondary forest age for each Brazilian biome. The dashed black lines represent the age threshold where more than 50% of secondary forests are accumulated.

Our dataset shows that a total of 262,791 km^2^ of secondary forests recovered in Brazil between 1986 and 2018 (Table 2). This corresponded to 59% of the area of old-growth forests deforested in the Brazilian Amazon between 1988 and 2019^29^. These secondary forests were distributed throughout the Brazilian territory, with the lower proportion in the Pantanal biome, contributing with 0.43% (1,120 km^2^) of the total area mapped and the highest proportion of 56.61% (148,764 km^2^) in the Amazon biome. The Caatinga biome accounted for 2.32% (6,106 km^2^) of the area of secondary forests in Brazil and had the youngest secondary forests, with more than 50% of the forests aged between 1 to 6 years. As expected, the Atlantic Forest biome, which is the second biome in terms of extent, covering 26.72% (70,218 km^2^) of the secondary forest area, had the oldest secondary forests, with more than 50% of the forests aged between 1 and 12 years (Fig. 5).

**Table 2:** Extent of the secondary forests area in each Brazilian biome in 2018.

Biome	Extent (km^2^)	Extent (%)
Amazon	148,764	56.61
Atlantic Forest	70,218	26.72
Caatinga	6,106	2.32
Cerrado	34,115	12.98
Pampa	2,469	0.94
Pantanal	1,120	0.43
Brazil	262,791	100

This dataset provides valuable information to support Brazilian climate change policies, such as the NDC^16^ submitted to the United Nations Framework Convention on Climate Change (UNFCCC) under the 2015 Paris Agreement. Under the NDC, Brazil intends to reduce the country’s greenhouse gas emissions by 43% below the 2005 levels in 2030. This is planned to be partially achieved by reaching zero illegal deforestation, as well as by restoring and reforesting 12 million hectares of forests policies by 2030^16^. Our dataset can, therefore, be used to identify areas of secondary forest growth and loss as well as identifying their age. This can help determine if Brazil’s NDC goal will be achieved. This dataset can also support the Brazilian Native Vegetation Protection Law^30^ (Law No. 12,651, of May 25, 2012), which enforces the restoration of forests within areas that suffered illegal deforestation in private properties. Finally, the dataset can also be used for environmental studies, including forest restoration, carbon emissions from forest fires, forest biomass estimation, carbon sequestration, among others.

To demonstrate the usefulness of our dataset, we calculated the potential net carbon uptake by secondary forests in each Brazilian biome between 1986 and 2018 through a pixel-by-pixel approach. For this estimate, we considered a linear net carbon uptake rate of 3.05 ± 0.19 Mg C ha^−1^ yr^−1^ (mean for the neotropical secondary forests)^9^ during the first 20-years of secondary forest succession, followed by a subsequent stabilization of the process, with a null growth^9,31,32^. Despite not considered in our estimates, it is important to highlight that carbon uptake rates vary among tropical secondary forests depending on climatic and environmental conditions^33^.

Adopting this method, we calculated that each secondary forest pixel (30-m spatial resolution or 0.09 ha of area) uptakes 0.275 ± 0.017 Mg C year^−1^, independent of the age, except for forests older than 20 years, which have a null carbon uptake rate, meaning that this forests have reached the climax stage, with the C gains from net primary productivity being offset by losses from heterotrophic respiration^31^. Applying this method to all secondary forest pixels in the age map of 2018, we estimated that stand secondary forests in Brazil were responsible for an uptake of 835 Tg C during the 33 years analysed (1986–2018) or 25.30 Tg C year^−1^ (Table 3). While the Pantanal biome had the lowest contribution, accounting for 0.42% of Brazil’s carbon uptake and stocking 3 Tg C in their secondary forests between 1986 and 2018, the Amazon biome had the largest contribution, accounting for 52.21% of the Brazilian secondary forest uptake. The cumulative secondary forest uptake in the Amazon biome during the period analysed led to the recovery of 436 Tg of the carbon that were lost from deforestation. Considering the period between 1988–2018, the estimated uptake by secondary forests in Brazil (784 Tg C) offsets 12% of carbon emissions from deforestation in the Brazilian Amazon alone (6,740 Tg C)^34^.

**Table 3:** Estimated cumulative net carbon uptake by secondary forests in each Brazilian biome between 1986 and 2018 (considering all stand secondary forests pixels in 2018).

Biome	Net Uptake (Tg C)	Net Uptake (%)
Amazon	436 ± 26.84	52.21
Atlantic Forest	260 ± 15.98	31.08
Caatinga	17 ± 1.03	2.01
Cerrado	111 ± 6.83	13.29
Pampa	8 ± 0.52	1.00
Pantanal	3 ± 0.21	0.42
Brazil	835 ± 51.40	100

The numbers in the second column are the net uptake values with the plus or minus signal representing the standard deviation of the estimations.

## Data Availability

All our codes are available from GitHub (https://github.com/celsohlsj/gee_brazil_sv) under the GNU General Public Licence v3.0^35^. In the GitHub repository users will find the freely available codes of our method and the Toolkit Download.
