# Author Correction: Benchmark maps of 33 years of secondary forest age for Brazil

**DOI:** 10.1038/s41597-020-00632-w

**Published:** 2020-08-28

**Authors:** Celso H. L. Silva Junior, Viola H. A. Heinrich, Ana T. G. Freire, Igor S. Broggio, Thais M. Rosan, Juan Doblas, Liana O. Anderson, Guillaume X. Rousseau, Yosio E. Shimabukuro, Carlos A. Silva, Joanna I. House, Luiz E. O. C. Aragão

**Affiliations:** 1 Tropical Ecosystems and Environmental Sciences lab – TREES, São José dos Campos, Brazil; 2 Instituto Nacional de Pesquisas Espaciais (INPE), São José dos Campos, Brazil; 3 University of Bristol, Bristol, United Kingdom; 4 Programa de Pós-graduação em Biodiversidade e Conservação, Universidade Federal do Maranhão (UFMA), São Luís, Brazil; 5 Laboratório de Ciências Ambientais, Centro de Biociências e Biotecnologia, Universidade Estadual do Norte Fluminense Darcy Ribeiro (UENF), Campos dos Goytacazes, Brazil; 6 University of Exeter, Exeter, United Kingdom; 7 Centro Nacional de Monitoramento e Alertas de Desastres Naturais (Cemaden), São José dos Campos, Brazil; 8 Programa de Pós-graduação em Agroecologia, Universidade Estadual do Maranhão (UEMA), São Luís, Brazil; 9 University of Maryland, College Park, United States of America; 10 University of Florida, Gainesville, United States of America

Correction to: *Scientific Data* 10.1038/s41597-020-00600-4, published online 14 August 2020

Following publication of this Data Descriptor it was found that the affiliation, *Programa de Pós-graduação em Agroecologia, Universidade Estadual do Maranhão (UEMA), São Luís, Brazil* was spelled incorrectly. This has now been corrected in both the HTML and PDF versions.

